# Retraction: A novel spectral transformation technique based on special functions for improved chest X-ray image classification

**DOI:** 10.1371/journal.pone.0351199

**Published:** 2026-06-23

**Authors:** The PLOS One Editors

The *PLOS One* Editors retract this article [1] due to concerns about potential manipulation of the publication process. These concerns call into question the validity and provenance of the reported results. We regret that the issues were not identified prior to the article’s publication.

The author did not agree with the retraction.

## References

[1] Aljohani A. RETRACTED: A novel spectral transformation technique based on special functions for improved chest X-ray image classification. PLoS One. 2025;20(6):e0325058. doi: 10.1371/journal.pone.0325058 40498709 PMC12157170

